# Availability, stock levels and usage of In-vitro diagnostics in the Bono region, Ghana: A cross-sectional study

**DOI:** 10.4102/phcfm.v15i1.4114

**Published:** 2023-10-23

**Authors:** Monica Ansu-Mensah, Desmond Kuupiel, Vitalis Bawontuo, Themba G. Ginindza

**Affiliations:** 1 Discipline of Public Health Medicine, School of Nursing and Public Health, University of KwaZulu-Natal, Durban, South Africa; 2 Health Economics and HIV and AIDS Research Division (HEARD), University of KwaZulu-Natal, Durban, South Africa; 3 Clinic, Sunyani Technical University, Sunyani, Ghana; 4 Department of Global Health and Sustainability, Faculty of Health Sciences, Durban University of Technology, Durban, South Africa; 5 Department of Health Services Management and Administration, School of Business, SD Dombo University of Business and Integrated Development Studies, Wa, Ghana; 6 Cancer and Infectious Diseases Epidemiology Research Unit (CIDERU), College of Health Sciences, University of KwaZulu-Natal, Durban, South Africa

**Keywords:** accessibility, stock level, funding, POC diagnostic testing, Bono Region

## Abstract

**Background:**

Point-of-care (POC) diagnostic tests play essential roles in diagnosis, surveillance, and disease management in health settings. Nevertheless, implementation challenges may hamper POC test accessibility. This study evaluated the availability and stock levels of the World Health Organization (WHO) prequalified existing in-vitro diagnostics (IVDs) for use in health facilities without laboratories.

**Aim:**

To evaluate the availability, stock levels, and usage of POC diagnostic tests.

**Setting:**

Bono Region, Ghana.

**Methods:**

This cross-sectional survey involved 102 randomly selected Community Health-based and Planning Services (CHPS), 12 district health depots, and a regional medical depot. Using a survey tool, data were collected on clinic staffing, availability and stock levels of tests, and funding sources. STATA 17 was employed for data analysis.

**Results:**

Majority (37.3%) of the respondents were community health nurses, with 4.4 mean years of work experience and 38 working hours per week. Of the 18 existing WHO prequalified POC tests for use at facilities without laboratories, 10 (56%), 2 (11%) and 0 (0%) were found at CHPS, regional, and district depots, respectively. Majority (183 out of 301) stock levels were low. Of the 10 available tests found, 7 scored 111 (36%) of ‘high use’. Supply chain management compliance was 5 (31%) out of 16. All CHPS received government funding with 25.5% of them receiving additional donor or internally generated funding.

**Conclusion:**

This study found poor supply chain management compliance, and low availability of POC tests in the Bono Region of Ghana.

**Contribution:**

The study outlines POC tests availability and usage in low-resourced setting.

## Introduction

Ghana, like other lower- and middle-income countries (LMICs), is faced with a double burden of communicable and non-communicable diseases.^1^ Despite this double burden of communicable and non-communicable diseases, the healthcare system is fragile and most of its health indicators are still far from reaching the target set by the United Nations Sustainable Development Goals (SDGs).^2,3,4^ For instance, maternal mortality is 310 per 100 000 live births, about 500% higher than the SDGs 2030 target of less than 70 per 100 000 live births, and neonatal mortality is 25 per 1000 live births.^5,6,7,8^ In light of this, Ghana is making efforts to extend healthcare services closer to the people through the establishment of Community-based Health and Planning Services (CHPS) at community level, and clinics and health centres at sub-district levels with a varied package of services including point-of-care (POC) diagnostic testing.^9,10,11,12,13,14,15,16,17,18,19,20^

Point-of-care diagnostic testing offers the healthcare professional the opportunity to administer diagnostic tests close to the patient and receive quick results.^21,22,23,24,25,26,27,28^ Point-of-care tests are medical devices, designed to meet the World Health Organization (WHO) ASSURED (affordable, sensitive, specific, user-friendly, rapid and robust, equipment-free, and deliverable to end user) quality standard.^12,27,29,30,31,32,33,34,35,36,37,38,39^ The introduction of the WHO Model List of an Essential In-Vitro Diagnostic (EDL) has been a remarkable strategy to reach and speed up a life-saving diagnosis, informing treatment and referral decisions and eliminating symptomatic treatment, particularly in health facilities without laboratories.^28,40,41,42^ The WHO EDL is additionally contributing to improving access to healthcare services and health outcomes in resource-limited settings.^42^ Notwithstanding the enormous benefits of POC testing services especially in healthcare facilities without laboratories, many challenges impede their successful implementation.^9,28^ For example, supply chain bottlenecks continue to hamper a sustainable stock level of POC diagnostics in low-resourced healthcare facilities.^43,44,45^ Moreover, POC diagnostic testing service implementation is bedevilled with funding challenges.^46^ There is a need to evaluate accessibility to POC testing services in resource-limited settings especially during this post-coronavirus disease 2019 (COVID-19) era to identify innovative solutions for sustaining POC testing services at health facilities without resourced laboratories in LMICs including Ghana.

In Ghana, little is known about POC diagnosis service provision at healthcare facilities without laboratories. Prior studies that evaluated the availability of POC testing in Ghana focused on Northern Ghana. Kuupiel et al. assessed the accessibility of pregnancy-related POC diagnostic services,^9^ as well as the supply chain management and stock levels of POC tests in the Upper East Region of Ghana.^47^ Ward et al. also investigated the availability and pricing of laboratory testing in the Northern Region to identify gaps regarding the WHO’s Essential Diagnostics List.^16^ Therefore, this study evaluated the availability and stock level of POC diagnostic tests at health facilities without laboratories (CHPS facilities) in the Bono Region of Ghana highlighting the healthcare providers’ experience and competencies, POC diagnostic test availability, stock levels, frequency of POC test usage, supply chain management, and funding. The authors hope the evidence provided by this study will inform the decisions of stakeholders such as healthcare professionals, the Government of Ghana (GOG), donors, POC diagnostic test manufacturers, and non-governmental organisations in health towards improving and bridging the POC testing accessibility gap.

## Research methods and design

### Study area

**FIGURE 1:**
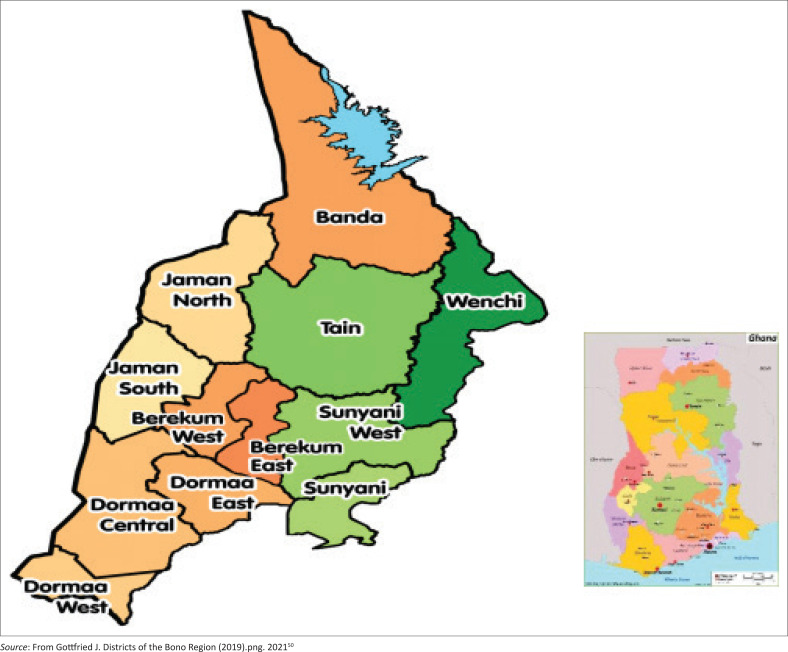
Map of Bono Region showing the 12 administrative districts.

The study was conducted in the Bono Region – one of the 16 regions of Ghana. The region was chosen because it is one of the newly created regions with few hospitals. The region has 12 administrative districts as shown in Figure 1. Sunyani is the regional capital. Each administrative district has a health depot, with 1 regional depot located in the regional capital, Sunyani, serving the 12 districts. The region lies in the mid-west of Ghana and shares local boundaries with the Bono East Region to the East, Savanna Region to the North, Ahafo Region, and the Western North Regions to the South. Internationally, the Bono Region shares a boundary with *La Cote d’Ivoire* to the West. The region covers about 11 481 sq.km area of land with a population of 1 208 965 with 49.4% male and 50.6% female.^48^ The region has 461 healthcare facilities including 306 CHPS, 50 clinics, 63 health centres, 21 maternity homes, 1 polyclinic, 2 psychiatry clinics, 1 regional hospital, 5 private hospitals, 5 Christian health hospitals, and 7 government hospitals.^49^

### Study design and study population

This is a cross-sectional quantitative study involving health facilities without laboratories (CHPS facilities), stores at the district health directorates, and the regional medical stores in the Bono Region of Ghana. The list of 306 CHPS which render POC diagnostic testing services in primary health care (PHC) facilities without laboratories, 12 stores at the health directorates, and 1 regional medical depot were obtained from the Bono Regional Health Directorate.

### Sampling

A list of 306 CHPS offering POC diagnostic testing services was stratified into 12 strata with each stratum representing one of the 12 administrative districts in the Bono Region. Using proportional sampling, 33% of the population from each stratum was selected. The use of proportional sampling allowed to obtain an accurate representative sample from each district. Finally, a simple random sampling technique was used to draw the total number of respondents from each stratum where all the names of facilities in each stratum (district) were written on pieces of paper, and the total number was selected from the stratum.

Additionally, all 13 depots were sampled through a census. This type of sampling helped extract data on all the depots in the population and avoided sampling errors for the study because there was a small population of depots.

### Sample size

Because a larger sample size will enable to have greater precision, 33% of CHPS, and all the district and regional health depots were selected. The formula


ni=n/N×Ni,

where *n* is the total sample size 1/3 × 306 = 102, where *N* is the total number of CHPS (*N* = 306), Ni is the number of CHPS in a district, and ni is the sample size taken from a district and n/N will be constant for every district. Therefore, *n/N* = 102/306 = 1/3 (Table 1). Hence, 102 CHPS, 12 district depots, and 1 regional depot were recruited for the survey. A total of 115 respondents including 3 physician assistants, 15 midwives, 17 general nurses, 38 community health nurses, 26 health assistants, 3 mental health officers, 12 district health depot managers, and 1 regional depot manager participated in the study.

**TABLE 1:** Distribution of sampled Community Health-based and Planning Services in this study.

Name of district	No. of CHPS (facilities without laboratories)	Sample size
Banda	8	3
Berekum East	20	7
Berekum West	10	4
Dormaa East	22	7
Dormaa Central	30	10
Dormaa West	13	4
Jaman North	28	9
Jaman South	40	13
Sunyani	35	12
Sunyani West	37	12
Tain	29	10
Wenchi	34	11

**Total**	**306**	**102**

### Data collection

Data were collected from June to July 2022 using the survey tool adopted by Kuupiel et al.^9^ The tool was pretested in 12 non-participating CHPS in the Bono Region and all necessary adjustments were made based on respondents’ feedback. For all participating CHPS, data were collected on human resource capacity (staffing); category of staff, and their competencies with the POC diagnostic test usage. Data were also taken on the availability and stock level of WHO in-vitro diagnostics (IVDs) for use in community settings and health facilities without laboratories from the district and regional depots, and the participated CHPS. The authors again collected data on the frequency of POC usage. Moreover, data were collected on inventory management variables such as bin cards, and control forms availability to determine compliance. Lastly, the authors collected data on funding to obtain the CHPS’s source of funding.

### Outcome measures

The primary outcomes of the study included the availability and stock levels of POC tests at the CHPS, districts and regional depots, and sources of funding for POC tests in the Bono Region. The secondary outcomes of this study were the frequency of POC test usage, compliance with supply chain management guidelines and inventory management at the PHC (CHPS) facilities.

### Data analysis

Availability of POC diagnostic tests was determined as follows: 13–18 high availability; 7–12 average availability, and 1–6 low availability. Responses on diagnostic POC test usage were analysed using 0% – 100% score scale, where 100% denotes ‘I do use’, and 0% ‘I do not use’. A stock of 100+, 50–99 and 1–49 was determined as high, average and low stock levels, respectively. Responses on the frequency of POC tests usage were analysed as: ‘more than once per day’; ‘daily’ ‘weekly’ ‘monthly’ and ‘once per year or less’ where POC tests are highly used, mostly used, fairly used, rarely used and does not used, respectively. A total of 90% – 100% was regarded as ‘compliant’ to supply chain management, demonstrating reliable and acceptable compliance with the specified guideline. A score less than 90% was regarded as ‘non-compliant’ to supply chain management, demonstrating unacceptable compliance of the CHPS to the specified guidelines. Data were processed in Microsoft Excel and exported into STATA statistical software of version 17 for all analyses. All processed data were grouped into frequencies, standard deviation (s.d.), and means with 95% confidence intervals for the responses.

### Ethical considerations

This study received ethical approval from the Ghana Health Service Ethics Review Committee with approval number, GHS-ERC:018/03/22 and the University of KwaZulu-Natal Biomedical Research Ethics Committee (BREC) with approval number, BREC/00004499/2022. The study obtained a gatekeeper permit from the Bono Regional Health Directorate, Sunyani, Ghana. All study participants agreed to participate by signing informed consent before the study.

## Results

### Respondents’ characteristics

Table 2a, Table 2b and Table 2c shows the characteristics of the selected CHPS and the respondents in this survey. The study recorded a 100% response rate from the staff in the selected CHPS facilities in the Bono Region. A total of 698 healthcare professionals were found in all 102 CHPS facilities. The largest proportion, that is, 234 (33.5%) were health assistants (nurse assistant clinical and nurse assistant preventive). Even though the evaluated CHPS were without laboratories, a minority of 3 (0.40%) respondents, were laboratory assistants. Respondents’ mean ± s.d. years of working experience and working hours per week were 4.43 ± 0.274 (95% CI: 3.89–4.97) and 37.76 ± 0.272 (95% CI: 37.23–38.30), respectively. Of the total 102 respondents from selected CHPS facilities, the largest proportion (38 = 37.3%) were community health nurses (nurse assistant preventive), and the least (3 = 2.9%) were Physician Assistants and Mental Health Officers. At the district and regional depots, 89 and 64 workers were found, respectively. All 12 district depot managers and 1 regional depot manager took part in the study.

**TABLE 2a:** Category of the 102 Community Health-Based and Planning Services surveyed in the Bono Region.

Staffing (*N* = 698) (Clinical staff)	Number of staff per category	Percentage per staff category	Range
Number of physician assistants	5	0.70	0–1
Number of midwives	117	17	0–5
Number of general nurses	75	11	0–4
Number of community health nurses	224	32	0–8
Number of health assistants	234	33.5	0–7
Number of laboratory assistants	3	0.40	0–3
Number of dispensary and/or pharmacy assistants	4	0.60	0–2
Others (mental health officers)	36	5	0–5

**TABLE 2b:** Category of the 102 Community Health-Based and Planning Services surveyed in the Bono Region.

Characteristics of respondents (*N* = 102)	Frequency	Proportion
Number of physician assistants	3	0.03
Number of midwives	15	0.14
Number of general nurses	17	0.17
Number of community health nurses	38	0.37
Number of health assistants	26	0.25
Others (mental health officers)	3	0.03

**TABLE 2c:** Category of the 102 Community Health-Based and Planning Services surveyed in the Bono Region.

Respondents’ details	Mean (s.d.)	95% Confidence Interval
Work experience (years)	4.43 ± 0.274	3.89 – 4.97
Working hours (week)	37.76 ± 0.272	37.23 ± 38.30

s.d., standard deviation.

### Availability, stock level and reasons for non-availability of point-of-care diagnostics at the regional medical stores

Of the total 18 existing IVDs in the WHO EDL recommended for implementation in health facilities without laboratories, 2 (11%) (HIV1 and 2 antibodies, and Plasmodium ssp. antigen tests) were available. The stock level of the HIV1 and 2 antibodies test was 56 700 while Plasmodium ssp. antigen test was 472 525. Two reasons were given for the non-availability of POC tests for glucose, haemoglobin A/c (Hb A/c), haemoglobin (Hb), urinalysis tests strips (UTS), human chorionic gonadotropin (HCG), hepatitis B surface antigen (Hep. B), anti-HCV antibody (Hep. C), combined HIV antibody/p24 Ag antigen (anti-HIV 24 Ag), qualitative HIV virological nucleic acid test (Qualitative HIV), and combined antibodies to T. Pallidum and HIV-1/2 (Anti-TP HIV1/2) are purchased from the open market. The remaining POC tests, such as Blood Typing, Albumin, Bilirubin, Ketones, Whole Blood Lactate (WBL), and CD4 Cell Enumerations (CD4 count) were reported as not testing sites for CHPS facilities.

### Availability, stock level,and reasons for non-availability of point-of-care diagnostics at district health directorate stores

The district depots recorded 100% non-availability of the existing POC diagnostic tests recommended by the WHO. It was reported that supplies were only made by the Regional Medical Stores through Ghana Integrated Logistics Management Information System. However, the districts are authorised to approve the CHPS facilities managers for collection of the programme logistics (HIV1/2 antibodies, and Plasmodium ssp. antigen), and to purchase from the open market where the test is allowed to be used at the site.

### Availability, stock level and reasons for non-availability of point-of-care diagnostics at the participated Community Health-based and Planning Services

Table 3 shows the availability, and stock level of POC diagnostic tests at the 102 CHPS facilities surveyed in the Bono Region of Ghana. Out of the 18 existing IVDs prequalified by the WHO to be used at health facilities without laboratories, 10 (56%) were available. The majority (93.1%) of the CHPS reported low (≤6 tests) availability of POC diagnostic tests, and 6.9% of the CHPS reported average (6–12 tests). The average number of POC tests available in the CHPS was 3 tests ± 2 (95% CI: 2.6–3.3). Glucose, Hb A/c, Hb, UTS, HCG, Hep. B, HIV1/2 antibodies, anti-HIV 24 Ag, Plasmodium spp. antigen and Anti TP HIV1/2 were the POC tests available in the CHPS surveyed. The study found varying stock levels in all the POC tests available. Majority 67% (183/301) of CHPS reported low, and 17% (52/301) reported average stock levels. However, Kobedi CHPS recorded the highest (1120) stock level for Plasmodium spp. antigen, while Amakyekrom CHPS, Namasa CHPS, and Botenso CHPS reported the lowest (2) each.

**TABLE 3:** Distribution of available point-of-care diagnostic tests, and stock levels for all Health-Based and Planning Services surveyed in the 12 districts (*N* = 102).

Name of the facility (CHPS)	Stock level of POC tests										
	Glucose	Hb A/c	Hb	UTS	HCG	Hep B	HIV1/2 antibodies	Anti-HIV 24 Ag	Plasmodium spp. antigen	Anti-TP HIV1/2	Total No. of Tests available at each CHPS
Sanwa	30	0	29	0	6	0	0	13	30	0	**5**
Wewa	0	0	0	0	10	0	14	0	0	0	**2**
Bofie	50	0	50	90	30	0	0	20	0	0	**5**
Amomaso	0	0	0	0	15	0	120	0	150	0	**3**
Benkasa	0	0	0	0	20	0	10	0	10	0	**3**
Nsapor	0	0	10	0	40	0	12	0	25	0	**4**
Kutre 1	0	0	28	40	34	0	0	0	150	32	**5**
Namasua	0	0	0	18	20	0	0	0	103	11	**4**
Senase	5	0	0	36	25	0	0	0	100	100	**5**
Fententaa	50	0	0	0	50	0	14	0	140	0	**4**
Nkyenkyeman	20	30	0	15	15	0	30	40	100	0	**7**
Ayinasu	0	0	0	0	10	0	0	0	100	0	**2**
Botokrom	25	50	20	0	30	0	0	50	500	0	**6**
Akrofro	0	0	0	30	30	0	0	0	9	80	**4**
Tewbabi/Abisaase	0	0	0	0	30	0	25	0	250	50	**4**
Mpatasie	0	0	6	8	50	0	0	0	0	50	**4**
Amankokwa	35	0	40	0	40	0	26	0	1000	12	**6**
BETCO	0	0	0	0	10	0	0	0	13	0	**2**
Kyereyawkrom	0	0	0	0	0	0	0	0	75	0	**1**
Asuotiano	0	0	0	0	0	0	0	50	0	0	**1**
Kyenkyenase	0	0	0	0	0	0	0	0	54	0	**1**
Akontanim	42	12	2	20	9	0	0	12	100	0	**7**
Meweremfiowuo	30	0	0	30	30	0	2	0	25	0	**5**
Yaw Barima	0	0	0	12	23	18	52	0	87	0	**5**
Kofibourshed	0	0	0	0	5	0	190	0	175	0	**3**
Amenfe	5	0	0	12	47	0	6	0	147	0	**5**
Manteware	0	0	0	0	30	0	0	0	25	0	**2**
Nsesereso	90	0	70	90	30	32	0	0	33	0	**6**
Antwirifo	0	0	0	0	7	0	0	0	0	0	**1**
Gonokrom	31	0	0	0	0	0	0	0	25	0	**2**
Atesikrom	0	0	0	0	15	0	50	0	125	0	**3**
Yawkrom K.K.K	0	0	0	0	11	0	7	0	29	0	**3**
Amakyekrom	0	0	0	0	19	0	0	0	5	0	**2**
Twumkrom	0	0	0	30	10	0	0	0	0	0	**2**
Asunsu	0	0	0	0	10	0	3	10	0	15	**3**
Tronang	0	0	0	0	14	0	0	0	0	0	**1**
Abonsrakrom	0	0	0	0	54	0	0	0	103	0	**2**
Nsenia	0	0	0	0	0	0	0	0	110	0	**1**
Yaw Owusu	0	0	30	3	13	49	0	0	0	5	**5**
Bonakire	0	0	0	0	0	0	0	0	200	0	**1**
Asiri	75	0	150	142	100	100	0	0	500	100	**7**
Goka Adenpranease	78	0	99	30	170	120	115	0	200	0	**7**
Adadiem	0	0	0	0	10	0	0	0	1100	0	**2**
Jamera	0	0	0	75	60	0	0	0	975	0	**3**
Asantekrom	0	0	0	0	0	0	0	0	250	0	**1**
Jenini	30	0	0	0	10	0	0	0	500	0	**3**
Asuokor	100	0	30	0	25	0	0	106	800	0	**5**
Nsonsomea	0	0	0	0	50	0	25	0	150	0	**3**
Kabile	0	0	0	0	0	0	0	0	800	0	**1**
Amanfoso	0	0	0	30	30	0	66	0	178	0	**4**
Yiadom (Suma)	25	14	25	0	15	4	200	0	475	0	**7**
Adiokor	0	0	0	0	50	0	200	0	249	0	**3**
Faaman	50	0	0	50	45	0	40	0	250	0	**5**
Katakyiekrom	0	0	0	0	0	0	0	0	750	0	**1**
Asempanaye	0	0	0	0	35	0	0	0	0	0	**1**
Tekese	0	0	0	50	60	0	8	0	275	0	**4**
Abuokrom	0	0	0	0	50	0	0	0	25	0	**2**
Miremano	25	0	0	100	50	0	100	0	100	100	**7**
Yaamensa	0	0	0	0	0	0	0	0	25	0	**1**
Konsia	0	0	0	0	2	0	0	0	0	0	**1**
Atuna	29	0	20	0	0	0	0	0	250	0	**3**
Tainano	0	0	0	0	50	0	0	0	300	0	**2**
Bodaa	0	0	0	0	55	0	0	0	0	0	**1**
Batea	0	0	0	0	0	0	0	0	0	0	**0**
Nkrankron	0	0	0	0	14	0	61	0	0	0	**2**
Wawasua	42	0	0	10	0	0	20	15	33	0	**5**
Watchman	0	0	0	0	26	0	0	0	53	0	**2**
Benu-Nkwanta	0	0	0	0	0	0	0	0	0	0	**0**
Kantro	0	0	0	0	0	0	0	0	75	0	**1**
Fiapre Zongo	0	0	0	10	10	0	0	0	27	0	**3**
Berlin top	0	0	0	0	0	0	0	0	0	0	**0**
Kobedi	30	0	25	0	20	0	0	0	1120	150	**5**
Obiri Yeboah	0	0	0	0	45	0	0	0	43	0	**2**
Abronye	0	0	0	25	50	0	20	0	0	0	**3**
Adantia	0	0	0	0	20	0	0	0	30	0	**2**
Addoe	0	0	0	0	20	0	0	0	40	0	**2**
Mantukwa	0	0	0	0	5	0	0	0	165	0	**2**
Twumasikrom	0	0	0	0	20	0	0	0	5	0	**2**
Ayakomaso	0	0	0	0	25	0	50	0	125	0	**3**
Akwasua	0	0	0	0	0	0	16	0	55	0	**2**
Yooyoso	0	0	0	0	0	0	0	0	0	0	**0**
Kwabenakuma	50	0	0	0	20	0	0	0	1000	0	**3**
Boreso	0	0	0	0	0	0	0	0	100	0	**1**
Tanom	0	0	37	0	25	0	0	0	85	0	**3**
Namasa	7	0	3	0	0	0	15	0	2	0	**4**
Hani	125	0	50	50	50	5	50	0	0	0	**7**
Nasana	0	0	0	0	4	0	0	100	0	0	**2**
Kyekyewere	0	0	0	0	25	0	0	0	25	0	**2**
Atomfoso	0	0	0	0	0	0	0	0	100	0	**1**
Tainso	0	0	0	0	0	0	15	0	300	0	**2**
Adamu	0	0	0	4	0	0	0	0	0	0	**1**
Tiadane	25	0	25	0	25	0	25	0	25	0	**5**
Njau-Tanoso	0	0	0	0	0	0	0	72	30	0	**2**
Ayigbe	0	0	0	0	0	0	25	0	50	0	**2**
Buoku	8	0	0	40	25	0	5	0	41	0	**5**
Wenchi Zongo	0	0	0	0	64	0	45	0	100	0	**3**
Botenso	3	0	0	0	4	0	0	0	2	0	**3**
Nwoase	0	0	0	0	3	0	48	0	0	0	**2**
Amponsakrom	0	0	0	0	0	0	0	0	108	0	**1**
Branam	0	0	0	0	0	0	25	0	100	0	**2**
Agubie	0	0	0	27	0	12	0	0	0	0	**2**
Nyinamponase	0	0	0	0	2	0	0	0	25	0	**2**

**Total CHPS with test**	**28**	**4**	**20**	**28**	**74**	**8**	**37**	**11**	**79**	**12**	-
**Total stock (*N* = 102)**	**1115**	**106**	**749**	**1077**	**2161**	**340**	**1735**	**488**	**16014**	**705**	-

CHPS, community health-based and planning services; HbA/c, heamoglobin A/c; Hb, haemoglobin; UTS, urinalysis test strips; HCG, human chorionic Gonadotropin; Hep. B, hepatitis B surface antigen; anti-HIV24Ag, combined HIV antibody/p24 Ag antigen; anti-TP HIV1/2, combined antibodies to T. Pallidum and HIV-1/2; No., number.

Two reasons were given for non-availability: not testing site, and inadequate funding. Majority 75% (Blood Typing, Ketone, Albumin, Bilirubin, WBL, and CD4 count POC tests) of the POC tests were reported as not testing sites for CHPS, and 25% (Hep. C, and Qualitative HIV) were not available due to inadequate funding.

### Frequency of point-of-care diagnostic test usage

Table 4 shows the frequency of POC diagnostic tests used for all tests found in the study. The largest proportion, that is, 111 (36%) of the CHPS reported ‘highly used’ (more than once per day) for 7 out of the 10 available POC diagnostic tests: Glucose, Hb, UTS, HCG, Hep. B, HIV1/2 antibodies and Plasmodium spp. antigen. Out of the 111 responses of ‘more than once per day’ from the various tests’ usage, Plasmodium spp. antigen recorded the majority, 66 (59.4%) while Hb recorded the least 0.9%. No POC test was recorded using ‘used once per year’.

**TABLE 4:** Distribution of frequency of usage of the available point-of-care tests found.

Name of test	No. of CHPS using test more than once per day	No. of CHPS using test once daily	No. of CHPS using test weekly	No. of CHPS using test monthly	No. of CHPS using test once per year
Glucose	3	9	11	5	0
Hb A/c	0	1	2	1	0
Hb	1	3	11	5	0
UTS	9	9	6	4	0
HCG	18	20	28	5	0
Hep. B	2	0	3	3	0
HIV1/2 antibodies	12	8	12	6	0
Anti-HIV 24 Ag	0	2	7	2	0
Plasmodium ssp. antigen	66	13	2	0	0
Anti-TP HIV1/2	0	0	8	4	0

CHPS, community health-based and planning services; HbA/c, heamoglobin A/c; Hb, haemoglobin; UTS, urinalysis test strips; HCG, human chorionic Gonadotropin; Hep. B, hepatitis B surface antigen; anti-HIV24Ag, combined HIV antibody/p24 Ag antigen; anti-TP HIV1/2, combined antibodies to T. Pallidum and HIV-1/2; No., number.

### Human resource performance and inventory management for the available point-of-care diagnostic tests at Community Health-based and Planning Services

Table 5 shows the compliance of CHPS to supply chain management guidelines in the Bono Region of Ghana. The study showed the CHPS had 5 (31%) out of 16 variables complying with supply chain management. Majority (92%) of users were trained on the existing POC diagnostic tests usage. Of the 102 CHPS surveyed, 80% had available standard operating procedure (SOPP) for stock (reagents) level management. However, the study found 24.5% and 23% of CHPS without approved written SOPP, and without SOPP for the safe disposal of existing POC tests respectively.

**TABLE 5:** Human resource performance, and compliance of community health-based and planning services to supply chain management guidelines in the Bono Region (*N* = 102).

Variable	*n*	%
**Human resource capacity**		
Users trained to use existing POC diagnostics appropriately	92	90.0
CHPS with an approved written standard operating procedure (SOPP) for performing each POC test	78	76.5
Availability of SOPP for stock (reagents) level management for personnel	82	80.0
Availability of SOPP for safe disposal of existing POC tests	79	77.5
**Inventory management**		
Availability of personnel whose duties include management of existing POC tests	80	78.0
Presence of an updated list of existing POC tests in the last 3 months	89	87.0
Document expiring dates of existing POC tests	79	77.0
Availability of document inventory levels for POC tests	95	93.0
Document unexplained losses (leakage) of POC diagnostic tests	85	83.0
Availability of storage facilities for test kits and diagnostic reagents	91	89.0
Availability of computerised or manually recorded inventory	95	93.0
Availability of basic records cards such as stock or bin cards	92	90.0
Availability of monthly consumption records	87	90.0
Availability of inventory control forms	87	85.0
Availability of expired POC diagnostic test	60	59.0
Compiled list of expired POC diagnostic tests	43	42.0

POC, point-of-care.

The majority (93%) of CHPS had computerised or manually recorded inventory, and 93% available document inventory levels for diagnostic POC tests. Again, 90% of the CHPS had basic records cards, as well as 90% had available monthly consumption records. All other inventory management activities scored less than 90% with as low as 59% of CHPS having expired POC tests including HIV1/2 antibodies, and anti-TP HIV1/2. Out of the 59%, majority (58%) of them had no compiled list of expired POC tests.

### Source of funding

**FIGURE 2:**
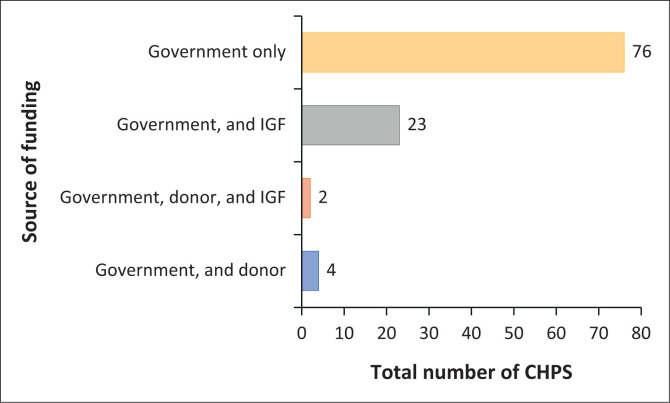
Distribution of source and period of funding of the 102 primary healthcare facilities surveyed.

Figure 2 shows the distribution of sources of funding for CHPS facilities in the Bono Region. All the CHPS facilities in the study are funded by the government. However, majority 76 (74.5%) of the CHPS facilities received government funding only; 23 (22.5%) received both government and internally generated funds (IGF); 4 (3.9%) received government and donor funding; and 2 (1.9%) received government, donor, and IGF.

## Discussion

Contributory factors facilitating accessibility to POC diagnostic testing services in resource-limited settings include the availability of the test, availability of funds, physical or geographical accessibility, affordability and religious and cultural factors.^51^ Therefore, the authors evaluated the accessibility of the WHO-recommended EDL tests for use in the PHC facilities in the Bono Region of Ghana focusing on availability, stock level, tests usage, human resource performance, inventory management and funding. The result of this study showed low availability of POC tests as well as stock level. The POC tests for Plasmodium spp. antigen and HCG were the most commonly available test used by most of the facilities surveyed. The study again revealed an uneven test availability in all the facilities across the 12 districts in the region. Moreover, variations exist in the facilities regarding stock levels of available POC tests. A maximum of 1120 and a minimum of 2 stock levels were found. All the CHPS facilities were funded by the government with some facilities benefiting from donor support. Even though IGF is supposed to be one of the major sources of funding, the research found only 25% CHPS on IGF. Additionally, the study recorded 44% non-availability of POC diagnostic tests including Blood Typing, Albumin, Bilirubin, Ketone, Hep. C, WBL, CD4 count and Qualitative HIV. Again, 89% of CHPS reported ‘more than once daily’, ‘daily’, and ‘weekly’ usage of POC tests. The findings from the district and regional depots also demonstrated that 33.3% of the tests were not to be used at PHC facilities. However, only Plasmodium spp. antigen and HIV1/2 antibodies (11.1%) were supplied and available at the regional depot while all the remaining tests are accessible from the open market. These findings are a wake-up call for quality improvement of POC diagnostic testing services in rural healthcare facilities towards health system strengthening and SDG 3.8 achievement of universal health coverage.^16,52,53^

Although POC tests like blood typing, albumin, bilirubin, ketone, Hep C, WBL, CD4 count and qualitative HIV were claimed as not testing sites for the CHPS, their impact of non-availability still holds. For instance, simple monitoring of antiretroviral therapy response for people living with HIV and/or AIDS will require referral to a higher-level facility for medications. Infants needing diagnosis of HIV infection will also face the same referral coupled with geographical accessibility challenges such as transport costs and its associated risks. Again, the non-availability of these tests means disease screening or monitoring ranging from blood grouping and rhesus factor type identification, kidney, liver, HCV, etc. disease investigation will require extra cost and time for the patient. This implies that out-of-pocket payment will be imposed on patients. Furthermore, CHPS facilities’ permission to purchase tests from the open market can expose them to corruption and result in expensive healthcare costs for innocent patients.

The results from the current study fully support the finding of a similar POC diagnostic testing survey in the Northern Region of Ghana.^16^ Wand et al.’s study equally revealed the low availability of POC diagnostic tests in the rural clinics of Northern Ghana.^16^ Their study further asserts and adds that plasmodium spp. antigen, HIV and HCG were the most used POC tests.^16^ Martin et al.’s study also reported the complexity of POC testing implementation in the LMICs.^54^ Similarly, Kuupiel et al.’s study reported low availability of POC diagnostic tests in Northern Ghana.^9^ Nonetheless, HCG, Hb and Hep B POC tests availability in Kuupiel et al. was higher than in the current study.^9^ The current study envisaged the important information on the accessibility of the WHO EDL in the PHC facilities within the resource-limited setting of Ghana to assist in the forthcoming POC diagnostic testing services implementation to help improve health outcomes. This study is the first of its kind to evaluate the availability of the existing WHO EDL for use in healthcare facilities without laboratories in rural settings in Southern Ghana. Moreover, this study could provide baseline data for future research into diagnostic testing services in PHC facilities based in low-resource settings. The study evaluated the current availability, stock level, use, human resource capacity, inventory management and funding for POC diagnostic testing services in the rural PHC CHPS in the Bono Region, which could help decision-makers and policy implementers in the region and Ghana as a whole.

However, the study has limitations despite its novelty and strength. The study failed to include other stakeholders, particularly the clients to assess their perspective of POC test accessibility. Therefore, clients’ experience with non-availability and access to the nearest referral facility in terms of physical distance, travel time and associated challenges are not known. The authors believe that the information provided in this research can assist decision-makers of POC testing services implementation to introduce a sustainable intervention to help strengthen access to POC testing in Ghana.

The 2021 Population and Housing Census report provides a guide to stakeholders with the policy development aim to ensure healthy lives for all. It states that ‘the report provides information for evidence-based decision-making to ensure the health and wellbeing of persons in Ghana and monitor progress towards the nation’s development goals’.^6^

Country prioritisation and level development of the WHO-recommended EDL are vital to help the accessibility of POC testing in the low-resource settings in Ghana.^39^ The low availability of POC diagnostic tests demonstrated in this study could affect Ghana’s pursuit to achieve SDG 3^55^ of ensuring healthy lives and promoting well-being at all ages as well as advancing universal health coverage.^56^ In this regard, it is important to provide available POC diagnostic tests to render accessible and effective POC diagnostics tests in a low-resource setting. To ensure a realistic availability of POC diagnostic testing in a resource-limited setting, physical accessibility, distance and travel time are crucial elements while adequate funding is necessary. The authors would, therefore, recommend a spatial study to assess POC testing accessibility particularly on the physical distance, and travel time to the nearest healthcare facility. Further studies could also evaluate the barriers/challenges, and potential solutions to POC testing accessibility in resource-limited settings, to ensure effective implementation of POC testing.

## Conclusion

Despite the limitations, this study is the first to assess the WHO model list of essential *in vitro* diagnostics in CHPS facilities in the Bono Region of Ghana. The study revealed low accessibility of POC diagnostic tests in the region because of poor availability and has ascertained the need to improve access to POC tests. Again, implementing POC tests such as Blood Typing, Albumin, Bilirubin, Ketone, Hep C, WBL, CD4 Count and Qualitative HIV will help eliminate some accessibility barriers. The availability of POC tests is the key barrier to the effective implementation of POC diagnostic testing in the low-resource settings of the Bono Region. The authors, therefore, encourage all major stakeholders including the GOG, Ministry of Finance, Ministry of Health, Ghana Health Service, donors and non-governmental organisations to help provide adequate funds as startup capital, procurement and supply of the needed POC tests in PHC facilities in the region. The authors recommend the WHO EDL as a standard for measuring the geographic access to POC tests with limited availability in the region. The authors again recommend a spatial study to evaluate the physical accessibility of POC tests in the PHC facilities in the region.

## References

[1] Roser M, Ritchie H, Spooner F. Burden of disease. Foot Ankle Int. 2018;39(3):387. 10.1177/1071100718756009 29406785

[2] Bygbjerg IC. Double burden of noncommunicable. Disea Preven. 2012;4:2003–2005. 10.1126/science.1223466 22997329

[3] Piot P, Caldwell A, Lamptey P. Addressing the growing burden of non-communicable disease by leveraging lessons from infectious disease management. J Glob Health. 2016;6(1):010304. 10.7189/jogh.06.010304 26955469 PMC4766788

[4] Rodriguez-Fernandez R, Ng N, Susilo D. The double burden of disease among mining workers in Papua, Indonesia: At the crossroads between Old and New health paradigms. BMC Public Health. 2016;16(1):951. 10.1186/s12889-016-3630-8 27609056 PMC5016925

[5] World Health Organization (WHO). WHO Ghana Bulletin. Accra: World Health Organization; 2021.

[6] Ghana Statistical Service (GSS). Ghana 2021 population and housing census: Fertility and mortality. Accra: Ghana Statistical Service; 2021.

[7] WHO. Building health systems resilience for universal health coverage and health security during the COVID-19 pandemic and beyond. Geneva: WHO; 2021, p. 1–52.

[8] MOH. Programme based budget estimates for 2021. Accra: Ministry of Health; 2021.

[9] Kuupiel D, Tlou B, Bawontuo V. Accessibility of pregnancy-related point-of-care diagnostic tests for maternal healthcare in rural primary healthcare facilities in Northern Ghana: A cross-sectional survey. Heliy. 2019;5(2):e01236-e. 10.1016/j.heliyon.2019.e01236 PMC6383048 30828664

[10] Sarfo B, Akweongo P, Mahama E. Factors associated with syphilis screening uptake among pregnant women in health facilities in Brong Ahafo Region of Ghana. Mate Health, Neonato Perina. 2015;1(1):1–11. 10.1186/s40748-015-0009-2 PMC4823680 27057324

[11] Ministry of Health. Ghana national healthcare quality strategy (2017–2021). Accra: Institute for Healthcare Improvement; 2016.

[12] Kuupiel Desmond BT, Bawontuo V. Poor supply chain management and stock-outs of point-of-care diagnostic tests in Upper East Region’s primary healthcare clinics, Ghana. PLoS One. 2019;14(2):1–15. 10.1371/journal.pone.0211498 PMC6392218 30811407

[13] Kuupiel D, Adu KM, Bawontuo V, Tabong PT, Adogboba DA, Mashamba-Thompson TP. Geographical access to point-of-care testing for hypertensive disorders of pregnancy as an integral part of maternal healthcare in Ghana. BMC Pregnancy Childbirth. 2020;20:1–9. 10.1186/s12884-020-03441-6 PMC7690122 33238918

[14] Kuupiel D, Adu KM, Bawontuo V. Estimating the spatial accessibility to blood group and Rhesus type point-of-care testing for maternal healthcare in Ghana. MDPI Diagn. 2019;9(175):1–14. 10.3390/diagnostics9040175 PMC6963207 31694228

[15] Gudu W, Addo B. Factors associated with utilization of skilled service delivery among women in rural Northern Ghana: A cross sectional study. BMC Pregnancy Childbirth. 2017;17(1):1–10. 10.1186/s12884-017-1344-2 28566088 PMC5452376

[16] Caleb L, Ward MZG, Timothy K. Amukele. Availability and prices of WHO essential diagnostics in laboratories in West Africa: A landscape survey of diagnostic testing in Northern Ghana. J Appl Lab Med. 2021;6(1):51–62. 10.1093/jalm/jfaa190 33438734

[17] Asamani JA, Ismaila H, Plange A. The cost of health workforce gaps and inequitable distribution in the Ghana Health Service: An analysis towards evidence – Based health workforce planning and management. Hum Resour Health. 2021;19:1–15. 10.1186/s12960-021-00590-3 33789670 PMC8010987

[18] Ansah EK, Narh-Bana S, Affran-Bonful H. The impact of providing rapid diagnostic malaria tests on fever management in the private retail sector in Ghana: A cluster randomized trial. BMJ. 2015;350:h1019. 10.1136/bmj.h1019 25739769 PMC4353311

[19] Amartey AO, Buaben KO, Mohammed A, Maru SM, Peeling R, Tengey S. Quality of malaria and HIV rapid diagnostic test kits (RDTs) in health facilities and medicines outlets in the Greater Accra Region of Ghana. Intern J Multidisc Res Anal. 2021;4(5):520–529.

[20] Adu-Gyasi D, Asante KP, Amoako S. Assessing the performance of only HRP2 and HRP2 with pLDH based rapid diagnostic tests for the diagnosis of malaria in middle Ghana, Africa. PLoS One. 2018;13(9):e0203524. 10.1371/journal.pone.0203524 30192839 PMC6128572

[21] Yager P, Domingo GJ, Gerdes J. Point-of-care diagnostics for global health. Ann Rev Biomed Eng. 2008;10:107–144. 10.1146/annurev.bioeng.10.061807.160524 18358075

[22] Vashist SK. Point-of-care diagnostics: Recent advances and trends. Biosensors. 2017;7(4):1013. 10.3390/bios7040062 PMC5746785 29258285

[23] Vafaie M, Biener M, Mueller M. Addition of copeptin improves diagnostic performance of point-of-care testing (POCT) for cardiac troponin T in early rule-out of myocardial infarction – A pilot study. Intern J Cardiol. 2015;198:26–30. 10.1016/j.ijcard.2015.06.122 26149334

[24] Uyoga S, George EC, Bates I. Point-of-care haemoglobin testing in African hospitals: A neglected essential diagnostic test. Br J Haematol. 2021;193(5):894–901. 10.1111/bjh.17431 33993492 PMC7611318

[25] Wang YC, Lee YT, Yang T. Current diagnostic tools for coronaviruses – From laboratory diagnosis to POC diagnosis for COVID-19. Bioeng Transl Med. 2020;5:e10177. 32838038 10.1002/btm2.10177 PMC7435577

[26] Gubala V, Harris LF, Ricco AJ, Tan MX, Williams DE. Point of care diagnostics: Status and future. Anal Chem. 2011;84(2):487–515. 10.1021/ac2030199 22221172

[27] Toskin I, Peeling RW, Mabey D. Point-of-care tests for STIs: The way forward. Sex Transm Infect. 2017;93(S4):S1–S2. 10.1136/sextrans-2016-053074 29223958

[28] Mashamba-Thompson TP. Diagnostics literacy advocacy model for vulnerable populations. MDPI Diagn. 2022;12(3):716. 10.3390/diagnostics12030716 PMC8946900 35328268

[29] Naseri M, Ziora ZM, Simon GP. ASSURED-compliant point-of-care diagnostics for the detection of human viral infections. Rev Med Virol. 2022;32(2):e2263. 10.1002/rmv.2263

[30] Smith S, Korvink JG, Mager D. The potential of paper-based diagnostics to meet the ASSURED criteria. RSC Adv. 2018;8(59):34012–34034. 10.1039/C8RA06132G 35548839 PMC9086909

[31] Pai NP, Vadnais C, Denkinger C. Point-of-care testing for infectious diseases: Diversity, complexity, and barriers in low- and middle-income countries. PLoS Med. 2012;9(9):e1001306. 10.1371/journal.pmed.1001306 22973183 PMC3433407

[32] Bengtson M, Bharadwaj M, Bosch AT. Matching development of point-of-care diagnostic tests to the local context: A case study of visceral leishmaniasis in kenya and uganda. Glob Health Sci Prac. 2020;8(3):549–565. 10.9745/GHSP-D-20-00028 PMC7541118 33008863

[33] Vandenberg O, Martiny D, Rochas O. Considerations for diagnostic COVID-19 tests. Nat Rev Microbiol. 2021;19(3):171–183. 10.1038/s41579-020-00461-z 33057203 PMC7556561

[34] Shephard M, Shephard A, Matthews S. The benefits and challenges of point-of-care testing in rural and remote primary care settings in Australia. Arch Pathol Lab Med. 2020;144(11):1372–1380. 10.5858/arpa.2020-0105-RA 33106858

[35] Wang S, Lifson MA, Inci F. Advances in addressing technical challenges of point-of-care diagnostics in resource-limited settings. Expert Rev Mol Diagn. 2016;16(4):449–459. 10.1586/14737159.2016.1142877 26777725 PMC4943866

[36] Fonjungo PN, Boeras DI, Zeh C. Access and quality of HIV-related point-of-care diagnostic testing in global health programs. Clin Infect Dis. 2016;62(3):369–374. 10.1093/cid/civ866 26423384

[37] Jaya Ziningi TPM-T. Lean and Agile point-of-care diagnostic services quality systems management for low- and middle-income countries. Poin of Care. 2016;15(4):152–157. 10.1097/POC.0000000000000111

[38] Mashamba-Thompson TP, Jama NA, Sartorius B. Implementation of point-of-care diagnostics in rural primary healthcare clinics in South Africa: Perspectives of key stakeholders. Diagn (Basel). 2017;7(1):3. 10.3390/diagnostics7010003 PMC5373012 28075337

[39] WHO. Second WHO model list of essential in vitro diagnostics. Who/Mvp/Emp/201905. Geneva: World Health Organization; 2019, p. 1–52.

[40] WHO. WHO publishes new essential diagnostics list and urges countries to prioritize investments in testing. Geneva: World Health Organization; 2021.

[41] Andriankaja OM, Muñoz-Torres FJ, Vergara JL. Utility of point-of-care vs reference laboratory testing for the evaluation of glucose levels. Diabet Med. 2019;36(5):626–632. 10.1111/dme.13922 30710457 PMC6599708

[42] Madimenos FC, Gildner TE, Eick GN, Sugiyama LS, Snodgrass JJ. Bringing the lab bench to the field: Point-of-care testing for enhancing health research and stakeholder engagement in rural/remote, indigenous, and resource-limited contexts. Am J Hum Biol. 2022;34(11):e23808. 10.1002/ajhb.23808 36166487

[43] Palmer T, Aiyenigba AO, Bates I. Improving the effectiveness of point of care tests for malaria and anaemia: A qualitative study across three Ghanaian antenatal clinics. BMC Health Serv Res. 2020;20(444):1–13. 10.1186/s12913-020-05274-7 PMC7238731 32429903

[44] Dassah ET, Adu-sarkodie Y, Mayaud P. Rollout of rapid point of care tests for antenatal syphilis screening in Ghana: Healthcare provider perspectives and experiences. BMC Health Serv Res. 2018;18(130):1–12. 10.1186/s12913-018-2935-y 29458363 PMC5819248

[45] Dassah ET, Adu-Sarkodie Y, Mayaud P. Estimating the uptake of maternal syphilis screening and other antenatal interventions before and after national rollout of syphilis point-of-care testing in Ghana. Int J Gynecol Obstetr. 2015;130(suppl. 1):S63–S69. 10.1016/j.ijgo.2015.04.013 25980367

[46] Boadu NY, Amuasi J, Ansong D. Challenges with implementing malaria rapid diagnostic tests at primary care facilities in a Ghanaian district: A qualitative study. Malar J. 2016;15(1):1–12. 10.1186/s12936-016-1174-0 26921263 PMC4769585

[47] Kuupiel D, Bawontuo V, Drain PK. Supply chain management and accessibility to point-of-care testing in resource-limited settings: A systematic scoping review. BMC Health Serv Res. 2019;3:1–11. 10.1186/s12913-019-4351-3 PMC6657084 31340833

[48] GSS. 2021 population and housing census: Presentation on general report Vol 3A to 3C 2022. Ghana: Ghana Statistical Service.

[49] Asare-tabi G. 2021 half year performance ranking Bono region. Sunyani: 2021.

[50] Gottfried J. Districts of the Bono Region (2019).png. Accra: 2021.

[51] Kuupiel D, Bawontuo V, Mashamba-thompson TP. Improving the accessibility and efficiency of point-of-care diagnostics services in low- and middle-income countries: Lean and Agile supply chain management. Diagnos MDPI. 2017;7(58):1–14. 10.3390/diagnostics7040058 PMC5745394 29186013

[52] Ankrah AK, Dako-Gyeke P. Factors influencing the delivery and uptake of early infant diagnosis of HIV services in Greater Accra, Ghana: A qualitative study. PLoS One. 2021;16(2):e0246876. 10.1371/journal.pone.0246876 33596241 PMC7888588

[53] Afulani PA. Determinants of stillbirths in Ghana: Does quality of antenatal care matter? BMC Pregnancy Childbirth. 2016;16(1):132. 10.1186/s12884-016-0925-9 27255155 PMC4891927

[54] Martin K, Wenlock R, Roper T. Facilitators and barriers to point-of-care testing for sexually transmitted infections in low- and middle-income countries: A scoping review. BMC Infect Dis. 2022;22(1):561. 10.1186/s12879-022-07534-9 35725437 PMC9208134

[55] United Nations. 2017 The 2030 agenda and the Sustainable Development Goals: An opportunity for Latin America and the Caribbean. New York: United Nations Development Programme; 2017.

[56] UNDP. 2018 Sustainable Develpment Goals 2030. Santiago: United Nations; 2018.

